# WRKY43 regulates polyunsaturated fatty acid content and seed germination under unfavourable growth conditions

**DOI:** 10.1038/s41598-017-14695-0

**Published:** 2017-10-27

**Authors:** Katja Geilen, Mareike Heilmann, Stefan Hillmer, Maik Böhmer

**Affiliations:** 1 Institut für Biologie und Biotechnologie der Pflanzen, Westfälische Wilhelms-Universität, Schlossplatz 7, Münster, Germany; 2 Institute of Biochemistry/Biotechnology, Martin-Luther University Halle-Wittenberg, Kurt-Mothes-Straße 3, D-06120 Halle, Germany; 3 Electron Microscopy Core Facility, University of Heidelberg, Im Neuenheimer Feld 345, 69120 Heidelberg, Germany

## Abstract

Seed germination and postgerminative growth of *Arabidopsis thaliana* and various other plant species are arrested in response to unfavourable environmental conditions by signalling events involving the phytohormone abscisic acid (ABA). In this study, we showed that loss of the seed-specific WRKY DNA-BINDING PROTEIN 43 (WRKY43) conferred increased tolerance towards high salt, high osmolarity and low temperature during seed germination in *Arabidopsis*. The *wrky43* loss of function lines displayed increased inhibition of seed germination in response to exogenous ABA; whereas lines overexpressing *WRKY43* were more tolerant towards exogenous ABA. Biochemical analysis of fatty acid composition revealed that loss of *WRKY43* increased polyunsaturated fatty acid content in seeds, particularly 18:2^Δ9,12^ and 18:3^Δ9,12,15^ in triacylglycerols and phospholipids, indicating an important physiological effect on fatty acid desaturation with ramifications for the tolerance of plants to cold and osmotic stress and possibly, for oilseed engineering. Molecular analyses showed that ABA-induced regulation of *FUSCA3*, *ZAT10* and seed storage proteins were absent in the *wrky43* mutant. In summary, *WRKY43* encodes for a novel positive regulator of ABA-dependent gene regulation and as a potent modulator of fatty acid desaturation and seed filling, which results in increased tolerance to abiotic stress.

## Introduction

*Arabidopsis* seed development is a complex process that is divided into two primary phases, embryo morphogenesis and maturation^1,2^. Embryo morphogenesis begins with double fertilization of the ovule by the pollen grain and ends with formation of embryonic and organ tissues. During the maturation phase, the embryo expands by accumulation of storage compounds, including 2S- (cruciferins) and 12S- (albumins) seed storage proteins (SSPs), triacylglycerols (TAGs) and starch^3^.

Abscisic acid (ABA) levels increase at the onset of seed maturation thereby inhibiting further embryonic growth and seed germination, resulting in primary dormancy and desiccation tolerance^4–8^. The LAFL network, a regulatory network consisting of the B3 domain transcription factors ABSCISIC ACID INSENSITIVE 3 (ABI3), FUSCA 3 (FUS3) and LEAFY COTYLEDON 2 (LEC2) and the HAP3 subunit of a CCAAT-binding protein complex, LEAFY COTYLEDON 1 (LEC1), controls major aspects of seed maturation from mid to late embryogenesis, including desiccation tolerance, primary dormancy, accumulation of seed storage compounds and embryo identity^1,9–13^. Expression of SSPs is partially suppressed in single *lec1*, *lec2*, *fus3* or *abi3* mutants and fully impaired in *lec1/abi3*, *fus3/abi3* and *lec2/fus3* double mutant seeds^12–15^. Moreover, expression of SSPs by FUS3 is ABA-dependent^12,16^. Disruption of *LEC1*, *LEC2* or *FUS3* leads to a reduced fatty acid (FAs) content and a change in FA composition, potentially via the transcriptional regulation of *FATTY ACID DESATURASE 2* (*FAD2*), *FAD3* and *FATTY ACID ELONGASE 1* (*FAE1*)^16–20^.

Storage reserve compounds provide nutrition for germination and seedling establishment. Germination, a process defined as the emergence of a part of the embryo through surrounding seed structures, is coupled with the continuation of embryonic growth^4,8^. The duration of the germination period is determined by water uptake (imbibition phase), which depends on seed properties such as seed coat permeability, seed size, dormancy state and the ratio of ABA to gibberellic acid (GA). Seed dormancy can be released by after-ripening, light and cold treatment (stratification)^4^.

Because ABA induces dormancy before germination, ABA DEFICIENT mutants *aba1* and *aba2* and the ABA INSENSITIVE mutant *abi1-5* show altered seed germination in response to ABA, in addition to a reduced primary dormancy^21–23^. Mutations in the *ABI1* and *ABI2* loci, encoding for PP2C protein phosphatases, members of the ABA core-signalling pathway, also lead to disrupted ABA responses. Seed maturation and developmental phenotypes, such as altered seed storage reserve accumulation, are not found in *aba2-1* mutant seeds; however, these seeds increase in seed size and seed mass^23–28^. Reactivating late embryogenesis programs and arresting the growth of germinating embryos^29^, the bZIP transcription factor ABI5 acts downstream of ABI3.

In this study, we address the contribution of WRKY DNA-BINDING PROTEINs (WRKY) to the regulation of the signalling network controlling the balance of stress responses and seed filling in *Arabidopsis*. WRKY proteins are a large transcription factor family, predominantly found in plants, with over 70 members in *A*. *thaliana*. WRKY proteins contain a conserved DNA-binding domain called the WRKY domain with circa 60 amino acids and the eponymous conserved amino acid sequence WRKYGQK and a C_2_H_2_ or C_2_HC zinc finger motif. WRKY transcription factors show high binding affinity to the W-box sequence TTGACT/C^30–32^. Whereas WRKY transcription factors have been mostly studied in response to biotic stress, some WRKY transcription factors are reported to have physiological roles in the control of abiotic stress responses. WRKY40 is directly regulated by ABA via subnuclear localization, and with ABA treatment, WRKY40 relocalizes from PHYTOCHROME B containing nuclear bodies to the nucleoplasm^33^. WRKY40, with other WRKY transcription factors, functions in seed germination^34,35^. Loss of function mutants of *WRKY2*, *WRKY40* and *WRKY63* are ABA-hypersensitive in seed germination^36–39^. By contrast, loss-of-function of *WRKY41* reduces sensitivity towards ABA during seed germination via direct regulation of *ABI3*, thereby reducing primary dormancy and thermoinhibition of germination^40^. WRKY46 and WRKY57 are reported to mediate drought tolerance^41,42^. In addition to these roles in mediating stress responses, WRKY transcription factors are also involved in the control of seed development. Mutation in *WRKY10/MINISEED3* reduces seed size by reduced endosperm growth and cellularization^43^. Disruption of *WRKY44/TRANSPARENT TESTA GLABRA 2* causes defects in seed coat pigmentation through impaired tannin and mucilage production^44,45^.

In this report, we identified the WRKY transcription factor WRKY43 as a negative regulator of ABA-inhibition of seed germination by screening a library of transcription factor overexpression lines for increased germination on ABA-containing medium. We further studied the role of WRKY43 in abiotic stress tolerance during seed germination. Microarray and RT-qPCR analyses revealed *FUS3*, SSPs and *ZAT10* as targets of transcriptional regulation by WRKY43. A physiological consequence of transcriptional misregulation in the *wrky43* mutant was elevated polyunsaturated fatty acid content, concomitant with increased tolerance to cold, salt and osmotic stress during seed germination.

## Results

### Identification of negative transcriptional regulators in ABA-dependent inhibition of seed germination

**Figure 1:**
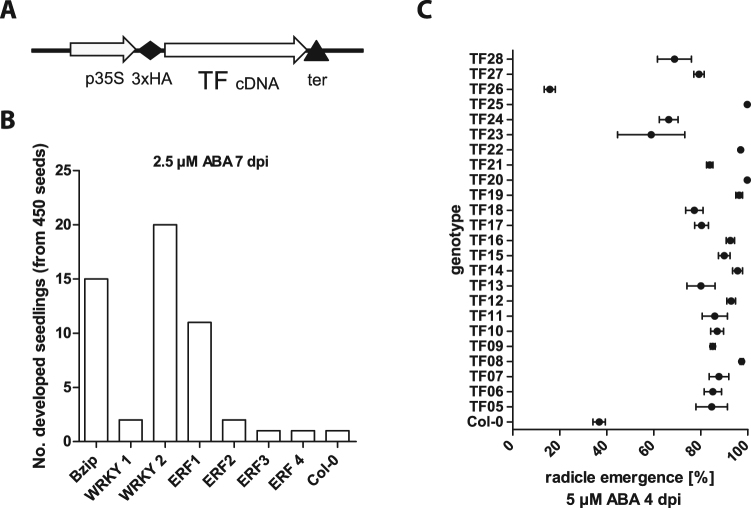
Screen of ORF overexpression transcription factor collections for impairment in ABA-dependent inhibition of germination. (**A**) Schematic illustration of ORF overexpression transcription factor collections. Transcription factors are expressed under 35S promoter and with an N-terminal HA-tag. (**B**) Number of developed seedlings of 450 seeds from each ORF overexpression transcription factor collection on 2.5 µM ABA-containing 0.5 MS medium after 7 days. (**C**) Radicle emergence (germination [%]) of OE-TF lines on 5 µM ABA-containing medium after 4 days. (mean ± SE; n = 3).

To identify transcription factors that negatively regulated ABA-dependent inhibition of seed germination, transgenic *A*. *thaliana* transcription factor overexpression lines (*At*TORF-Ex) were screened for impairment of ABA-inhibition of seed germination. Seven *At*TORF-Ex collections, each containing pooled populations of seeds, which each contain one of approximately 30 bZIP, WRKY and ETHYLENE RESPONSE FACTOR (ERF) transcription factors^46,47^ as an overexpression construct, were tested (Fig. 1A). From each collection, 450 seeds were screened for cotyledon greening after 7 dpi on 0.5 MS media containing 2.5 µM ABA. Fifteen seeds of the bZIP pool, 20 seeds of the second WRKY pool and 11 seeds of the first ERF pool developed into green seedlings under conditions in which only one of the Col-0 wild-type seeds germinated (Fig. 1B). By transferring green seedlings to soil, 28 overexpression transcription factor lines (TF1-TF28) were isolated. Rescreening of the descendants of these lines for radicle emergence after 4 dpi on 5 µM ABA-containing 0.5 MS medium confirmed increased germination rates on ABA-containing medium for 23 lines, whereas one line showed reduced germination, and four lines did not germinate (Fig. 1C). A total of 23 OE-TF lines were established as negative regulators of ABA-dependent inhibition of seed germination. After amplification of the open reading frames and sequencing using primers against the CaMV 35S promoter, line OE-TF17 was identified to contain transcription factor *WRKY43*.

To determine whether other ABA-induced physiological responses were also affected by overexpression of WRKY43, we tested water loss from detached rosettes (Figure S1A), and no apparent differences were detected in water loss comparing TF17 (OE-*WRKY43*) and Col-0.

### Molecular characterization of a *wrky43* transposon insertion mutant

**Figure 2:**
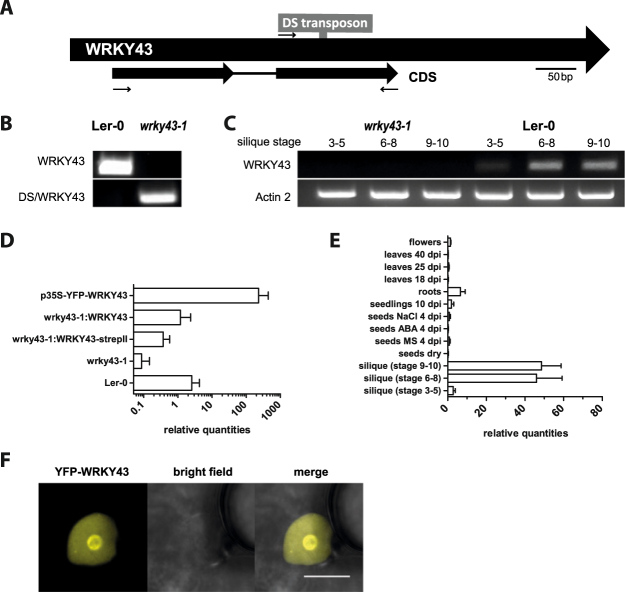
Characterization of *wrky43-1* mutant (ET5604) and WRKY43 expression analysis. (**A**) *wrky43-1* with transposon insertion in the second exon. Exons (black) and intron (black line) are indicated. Two pairs of primers (43_f and 43_rev; DS and 43_rev) were used to check transposon-insertion and are indicated with arrows. (**B**) PCR screen for transposon insertion in Ler-0 wild type and *wrky43-1* mutant. (**C**) RT-PCR of Ler-0 wild type and *wrky43-1* mutant displayed no full length WRKY43 transcript in *wrky43-1* silique cDNA. Actin 2 was used as the control. (**D**) RT-qPCR of *WRKY43* transcript levels in Ler-0 wild type, *wrky43-1* mutant, complementation lines *wrky43-1*:*WRKY43* and *wrky43-1*:*WRKY43* *-strepII* and overexpression *WRKY43* line p35S-*YFP-WRKY43* (mean ± SE; n = 3). (**E**) Expression profiles of *WRKY43* in various *A*. *thaliana* tissues. *WRKY43* expression was detectable in silique stages 6–10 and in roots (mean ± SE; n = 3). (**F**) Confocal images of YFP-WRKY43 in transiently transformed *N*. *benthamiana* leaves (scale bar = 10 µm).

To further study the function of WRKY43, the only available loss of function mutant, a homozygous *Arabidopsis wrky43-1* mutant line in Ler-0 background (ET5604), carrying a transposon insertion in the second exon was isolated (Fig. 2A). RT-PCR and RT-qPCR verified expression of *WRKY43* in Ler-0 wild-type controls, whereas in the *wrky43-1* line, no *WRKY43* transcript was detected (Fig. 2B–D). Adult *wrky43-1* mutant plants had no obvious morphological or developmental phenotypes. The *wrky43-1* mutant line was complemented with a genomic fragment, encompassing *WRKY43* promoter and gene, with (*wrky43-1*:*WRKY43-strepII*) and without a C-terminal StrepII-tag (*wrky43-1*:*WRKY43*), both of which largely restored WRKY43 expression. Additionally, a line overexpressing *WRKY43* under the CaMV 35S promoter, N-terminally tagged with YFP (*35S:YFP-WRKY43*), was generated in the Ler-0 background, resulting in a line with an increased level of *WRKY43* expression (Fig. 2D).

### *WRKY43* is expressed during seed maturation

*WRKY43* was primarily expressed during silique stages 6-10 of seed development and was expressed to a lesser extent in roots (Fig. 2E). Therefore, WRKY43 was primarily expressed in early- and mid-seed maturation phases, which was consistent with public microarray data in which *WRKY43* was primarily expressed during seed stages 7 and 8^48^. Subcellular localization was tested by transiently transforming *N*. *benthamiana* leaves, and YFP-WRKY43 was specifically localized to the nucleus and the nucleolus (Fig. 2F). In contrast to WRKY18 and WRKY40, no nuclear body localization was observed^33^.

### Disruption of *WRKY43* alters seed germination on ABA, NaCl and mannitol

**Figure 3:**
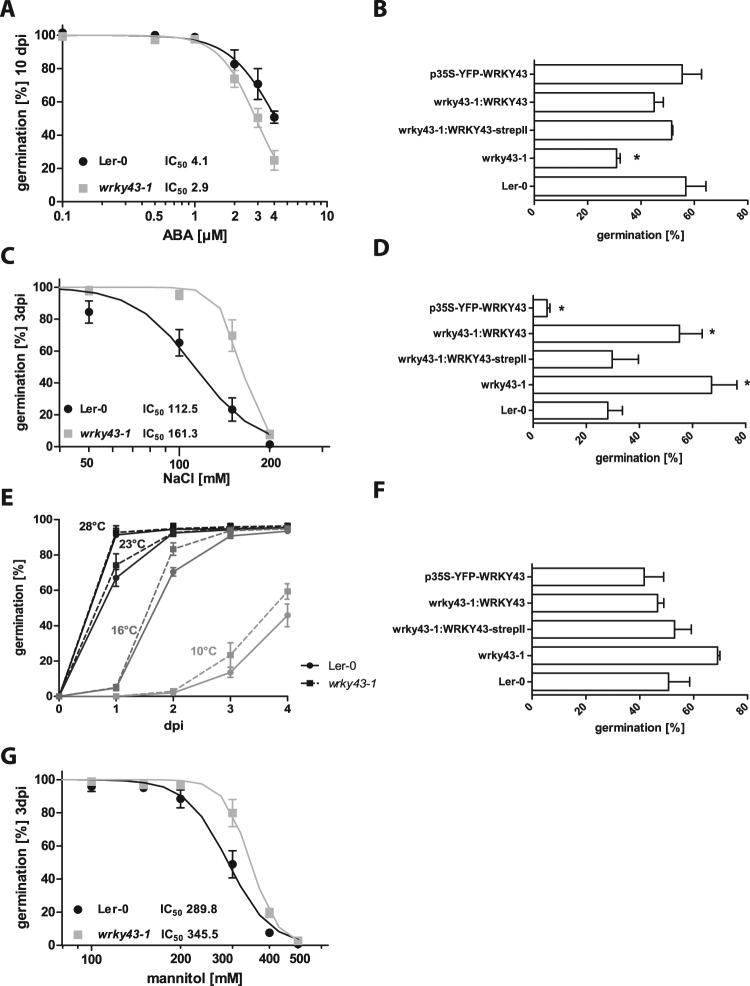
Disruption of *WRKY43* decreases germination on ABA but increases tolerance to NaCl, cold and mannitol. (**A**) Germination (radicle emergence) of Ler-0 *and wrky43-1* after-ripened seeds after 10 days incubation on increasing ABA concentrations. Data were normalized and fitted (log(inhibitor) vs. normalized response, Variable slope) to calculate IC_50_ values (mean ± SE, n = 3). (**B**) Germination (radicle emergence) of after-ripened seeds after 10 days incubation on 4 µM ABA-containing 0.5 MS medium (mean ± SE, n = 3). Asterisks indicate significant differences using an unpaired t-test (p < 0.05). (**C**) Germination (radicle emergence) of Ler-0 *and wrky43-1* after-ripened seeds after 3 days incubation on increasing NaCl concentrations. Data were normalized and fitted (log(inhibitor) vs. normalized response, Variable slope) to calculate IC_50_ values (mean ± SE, n = 3). Asterisks indicate significant differences using an unpaired t-test (p < 0.05). (**D**) Germination (radicle emergence) of after-ripened seeds after 3 days incubation on 150 mM NaCl-containing 0.5 MS medium (mean ± SE, n = 3). Asterisks indicate significant differences using an unpaired t-test (p < 0.05). (**E**) Germination (radicle emergence) of Ler-0 an*d wrky43-1* after-ripened seeds after 4 days incubation on 0.5 MS medium at decreasing temperatures from 28 °C to 10 °C (mean ± SE, n = 3). (**F**) Germination (radicle emergence) of after-ripened seeds after 4 days incubation on 0.5 MS medium at 10 °C (mean ± SE, n = 3). (**G**) Germination (radicle emergence) of Ler-0 and *wrky43-1* after-ripened seeds after 3 days incubation on increasing mannitol concentrations. Data were normalized and fitted (log(inhibitor) vs. normalized response, Variable slope) to calculate IC_50_ values (mean ± SE, n = 3). Asterisks indicate significant differences using an unpaired t-test (p < 0.05).

In the reverse genetic screen, *WRKY43* was identified to encode a negative regulator of ABA-inhibition of seed germination. Therefore, after-ripened seeds of *wrky43-1* mutant line and Ler-0 wild-type line were germinated on 0.5 MS medium supplemented with increasing concentrations of ABA to test for altered ABA sensitivity. The *wrky43-1* mutant showed increased sensitivity towards ABA. In the presence of 4 µM ABA and after 10 days, about 50% of Ler-0 seeds germinated, whereas only about 25% of the *wrky43-1* mutant seeds germinated (Fig. 3A). For *wrky43-1*, the half maximal inhibitory concentration (IC_50_) of ABA was reduced by nearly 30% compared with that determined for Ler-0. Complementation of the *wrky43-1* mutant restored germination rates to wild-type levels. In contrast to the initial genetic screen in the Col-0 background, overexpression of YFP-tagged *WRKY43* in the Ler-0 background did not increase seed germination as compared with Ler-0 (Fig. 3B). *Arabidopsis thaliana* ecotypes Ler-0 and Col-0 had very different ABA responsiveness. Therefore, we also tested loss of function of *WRKY43* in ecotype Col-0 using an RNAi-*WRKY43* construct. *WRKY43* loss of function reduced seed germination in the presence of 10 µM ABA to around 32% compared with the around 60% in Col-0 (Figure S2).

To delineate the role of WRKY43 in the control of primary seed dormancy, germination of freshly harvested seeds on water-agar plates was tested with and without prior stratification for 3 days at 4 °C (Figure S1B). No differences were observed between *wrky43-1* mutant and wild-type seeds under the tested conditions. Furthermore, expression levels of DELAY OF GERMINATION 1 (DOG1), a key regulator of seed dormancy^49,50^, displayed no transcriptional differences compared with wild-type plants (Figure S1C). Taken together, these results indicated that loss of *WKRY43* did not affect primary dormancy of seeds. Seed size measurements of water-imbibed seeds revealed no significant differences in the seed size enlargement due to physical water uptake (Figure S1D). Mucilage stained with ruthenium red revealed no detectable differences in the seed covering mucilage layers of *wrky43-1* seeds compared with those of the wild type (Figure S1E). Because of the expression of *WRKY43* in roots, we also tested root elongation on ABA-containing medium with *wrky43-1* mutants but detected no substantial differences (Figure S1F). No morphological differences between *wrky43-1* seeds and wild-type (Ler-0) seeds were observed in dissected embryos and seed coats (4-10 days after flowering) (Figure S1G) during germination (Figure S1H) or by scanning and transmission electron microscopy (Figure S1I,J).

Taken together, these data indicated that WRKY43 had a unique role as a negative regulator in controlling ABA-inhibition of seed germination but had no effect on other ABA responses or on primary seed dormancy.

To test whether WRKY43 affected seed germination also in response to other abiotic stresses, germination on 0.5 MS medium was tested with increasing concentrations of NaCl and mannitol and under cold temperature (10 °C) (Fig. 3C–G). The *wrky43-1* mutant displayed an increase in seed germination on 150 mM NaCl from about 31% to about 73%. Concomitantly, the IC_50_ value for NaCl increased by more than 40% for the *wrky43-1* mutant compared with the wild-type control (Fig. 3C). However, overexpression of *WRKY43* resulted in decreased germination rate on medium containing 150 mM NaCl, from around 28% for the wild type to around 5%. Complementation of *wrky43-1* mutant plants with *WRKY43* with and without a StrepII-tag reduced germination rates from around 67% for the mutant to around 55% and 39%, respectively (Fig. 3D). Furthermore, germination at 10 °C improved for *wrky43-1* mutant seeds at around 69% compared with about 50% for Ler-0 seeds (Fig. 3E). Complementation lines showed similar germination rates to those of Ler-0 wild type (Fig. 3F). Germination on 300 mM mannitol also increased for *wrky43-1* mutant seeds, up to around 77% compared with Ler-0 at about 46%, and the IC_50_ value increased for *wrky43-1* by around 20% compared with that of the wild type (Fig. 3G).

To summarize, disruption of *WRKY43* led to decreased germination rates on ABA and simultaneously increased tolerance to salt, osmotic and cold stress during germination. This contrasting effect on germination under different abiotic stress conditions, distinguishes *wrky43-1* from mutants with defects in ABA signalling that displayed an increased sensitivity to ABA and salt during germination^51,52^.

### Disruption of *WRKY43* increases polyunsaturated fatty acid content in TAGs and phospholipids of seeds

**Figure 4:**
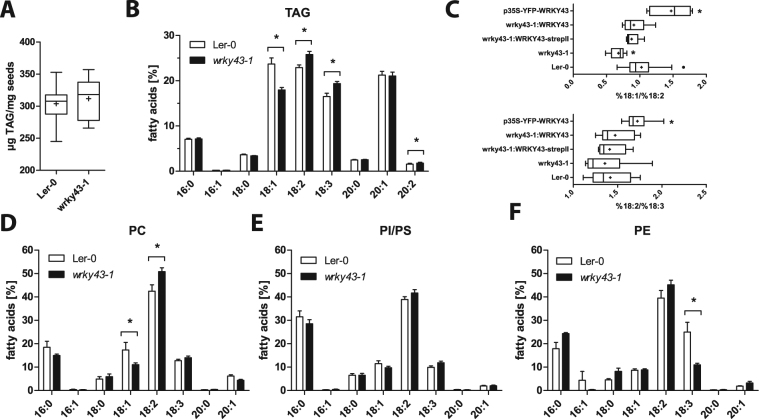
Disruption of *WRKY43* increases polyunsaturated fatty acid content in TAGs and phospholipids. (**A**) Total fatty acid content of dry seeds. Data are shown as box plots with Tukey whiskers, with plus signs indicating means. (**B**) Triacylglycerol (TAG) fatty acid composition of dry seeds. Statistical analysis was performed with one-way ANOVA (p < 0.05) and Bonferroni posttest; asterisks indicate significant differences compared with the wild type. (**C**) %18:1/%18:2 desaturation rate (FAD2 activity) and %18:2/%18:3 desaturation rate (FAD3 activity). Data are shown as box plots with Tukey whiskers, with plus signs indicating means. Statistical analysis was performed with one-way ANOVA (p < 0.05) and Bonferroni posttest; asterisks indicate significant differences compared with the wild-type Ler-0. (**D**) Fatty acid composition of phosphatidylcholine (PC). (**E**) Fatty acid composition of phosphatidylinositol (PI) and phosphatidylserine (PS). (**F**) Fatty acid composition of phosphatidylethanolamine (PE). Fatty acid composition of phospholipids in 8 DAF old seeds of Ler-0 and *wrky43-1* mutant. Data are shown as bar charts. Statistical analysis was performed with one-way ANOVA (p < 0.05) and Bonferroni posttest; asterisks indicate significant differences compared with Ler-0 wild type.

The phenotypes observed for the *wrky43-1* mutant, namely, the increased tolerance to NaCl, mannitol and cold during seed germination, resembled phenotypes described for *fad2* and *fad3* mutant plants^53,54^, suggesting that the effects might be related to fatty acid metabolism, a crucial step during seed development. Therefore, content and quality of seed fatty acids were determined. Ler-0, *wrky43-1* and complementation lines were grown in parallel, and the total fatty acid content of dry seeds was analysed by gas chromatography with flame ionization detector (GC-FID) (Fig. 4). The total fatty acid content was unchanged compared with wild-type seeds. However, the fatty acid composition of *wrky43-1* mutant seeds differed significantly from that of wild-type Ler-0 seeds. The percentage of oleic acid (18:1^Δ9^) was reduced from 24% for Ler-0 to 18% for *wrky43-1*, whereas the percentages of polyunsaturated fatty acids increased from 23% for Ler-0 to 26% for *wrky43-1* for linoleic acid (18:2^Δ9,12^) and from 17% for Ler-0 to 19% for *wrky43-1* for linolenic acid (18:3^Δ9,12,15^) (Fig. 4A,B).

The ratios of 18:1 to 18:2 and 18:2 to 18:3 fatty acids were calculated, to analyse the proportion of fatty acid desaturation by ω-6 and ω-3 desaturases. The 18:1/18:2 ratio decreased significantly for the *wrky43-1* mutant, whereas overexpression of *WRKY43* resulted in a significant increase of the 18:1/18:2 ratio. Complementation lines showed no significant differences compared with the wild type (Fig. 4C). Similar results were obtained for the 18:2/18:3 ratio, although these alterations were much smaller than those of the 18:1/18:2 ratios (Fig. 4C). These results indicated a significant effect of up- or down-regulation of *WRKY43* expression on ω-6 desaturation during seed filling.

In addition to the analysis of total seed fatty acids of dry mature seeds, which reflected mostly their TAG content, developing seeds were also analysed 8 DAF for the fatty acid composition in phospholipids. The percentage of polyunsaturated FAs in TAGs increased by 6% in *wrky43-1* mutants in developing seeds. Loss of *WRKY43* increased the percentage of linoleic acid and linolenic acid for phosphatidylcholine (PtdCho) by 12%, for phosphatidylinositol (PtdIns) and phosphatidylserine (PtdSer) by 5% and for phosphatidylethanolamine (PtdEtn) by 10% (Fig. 4D–F).

To determine whether WRKY43 had a specific role as a regulator of omega-3 and omega-6 fatty acid content in seeds, the fatty acid composition was also measured for phospholipids in leaves of 4-week-old plants (Figure S3C). In this material, no differences among wild type, *wrky43* mutant and WRKY43 overexpression lines were detected, indicating that effects of WRKY43 on the regulation of fatty acid desaturation were seed specific.

FAD enzymes mediate the conversion of saturated to unsaturated fatty acids by insertion of double bonds to the carbon chain (Figure S3A). We hypothesized that the increased proportion of unsaturated fatty acids in the *wrky43-1* mutant could be caused by a regulatory effect of WRKY43 on the expression of FADs. Several FAD enzymes relevant for the conversion of the fatty acids were changed in the *wrky43-1* mutant plants, including the ER localized FAD2 and plastid-localized FAD6 and two delta12 desaturases that catalyse the synthesis of linoleic acid. Further synthesis of linolenic acid by double bond insertion was induced by the delta15 desaturases FAD3 (ER localized), FAD7 and FAD8 (both plastid localized). To test a possible effect of WRKY43 on the expression of *FAD2*, *FAD6*, *FAD3*, *FAD7* and *FAD8*, quantitative RT-PCR experiments were performed (Figure S3B). In summary, no statistically significant changes were observed in FAD transcription levels for *wrky43-1* mutant developing seeds and for seeds overexpressing *WRKY43* that could account for the effects on the fatty acid patterns described above.

In conclusion, increased tolerance to salt, osmotic and cold stress of the *wrky43-1* mutant might be attributed to an increased proportion of unsaturated fatty acids. Increased desaturation of membrane lipids improves membrane fluidity, although the molecular nature of increased lipid desaturation by WRKY43 remains unclear.

### Disruption of *WRKY43* inhibits ABA-dependent up-regulation of *FUS3* and seed storage protein gene expression

**Figure 5:**
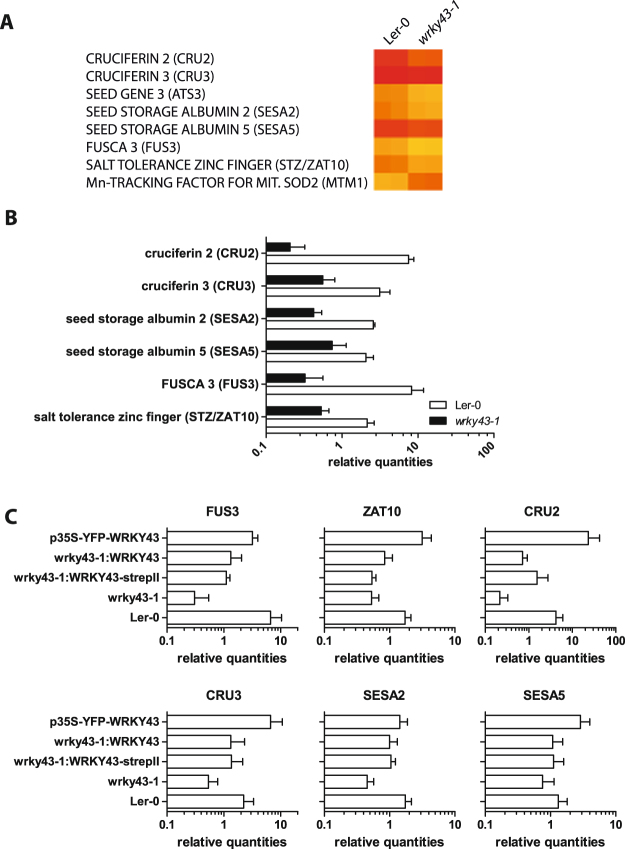
Disruption of *WRKY43* reduces transcription levels of seed storage proteins, *FUS3* and *ZAT10*. (**A**) Microarray analyses of seeds treated for 4 days with 2 µM ABA (n = 2) for certain significant differently expressed genes. (**B**) Expression profiles of SSPs, *FUS3* and *ZAT10* in Ler-0 wild type and *wrky43-1* mutant seeds treated with 2 µm ABA for 4 days. RT-qPCR data are shown as bar charts with a logarithmic scale (mean ± SE, n = 3). (**C**) Expression profiles of SSPs, *FUS3* and *ZAT10* in Ler-0 wild type, complementation and *WRKY43* overexpression seeds treated with 2 µM ABA for 4 days. RT-qPCR data are shown as bar charts with a logarithmic scale (mean ± SE, n = 3).

To identify target genes of WRKY43, microarray experiments from two biological replicates of seed batches 4 days after incubation on 2 µM ABA-containing medium were performed using an Affymetrix Aragene 1.0 st array. Differential expression analysis between *wrky43-1* and Ler-0 identified several differentially regulated genes with significant regulation (FC > 2, p < 0.01). The expression levels of *WRKY43* were too low for detection by microarray. Seed storage proteins, in contrast to the storage lipid synthesis genes, were significantly repressed under the experimental conditions. The storage proteins in *Arabidopsis* seeds are represented by the 12S albumins CRUCIFERIN 1 (CRU1), CRU2, CRU3 and At1g03890 and the 2S albumins 2S1 to 2S5^3^. Microarray data indicated that all cruciferins and *2S2* and *2S5* were significantly down regulated (Fig. 5A). Moreover, *FUS3*, a member of the LAFL network, and the *SALT TOLERANCE ZINC FINGER* (*ZAT10/STZ*), a salt-induced zinc-finger protein^15,55^, were significantly down regulated in *wrky43-1* mutant seeds. RT-qPCR confirmed regulation of seed storage proteins *CRU2*, *CRU3*, *SESA2* and *SESA5* and *FUS3* and *ZAT10* in ABA-treated seeds (Fig. 5B). Complementation of *wrky43-1* was sufficient to largely restore SSP, *FUS3* and *ZAT10* expression compared with Ler-0 wild type, whereas overexpression of *WRKY43* increased SSP and *ZAT10* expression (Fig. 5C). To determine ABA-dependency of this regulation, transcription levels of SSPs, *FUS3* and *ZAT10* were also measured in developing seeds (whole siliques), dry seeds, seeds 4 days after incubation on 0.5 MS medium and seeds 4 days after incubation on 150 mM NaCl-containing medium (Figure S4). *FUS3* and seed storage protein expression was only affected in response to ABA and to NaCl. By contrast, *ZAT10* regulation was also apparent in silique stages 9-10 in the absence of ABA in which *ZAT10* expression was repressed in *wrky43-1* mutant. These data indicated that WRKY43 played an ABA-dependent role in regulating *FUS3* and seed storage proteins, whereas *ZAT10* was also differentially regulated in the absence of external stimuli such as ABA or NaCl.

### Transcription factor WRKY43 directly regulates *ZAT10* but not *FUS3* gene expression

**Figure 6:**
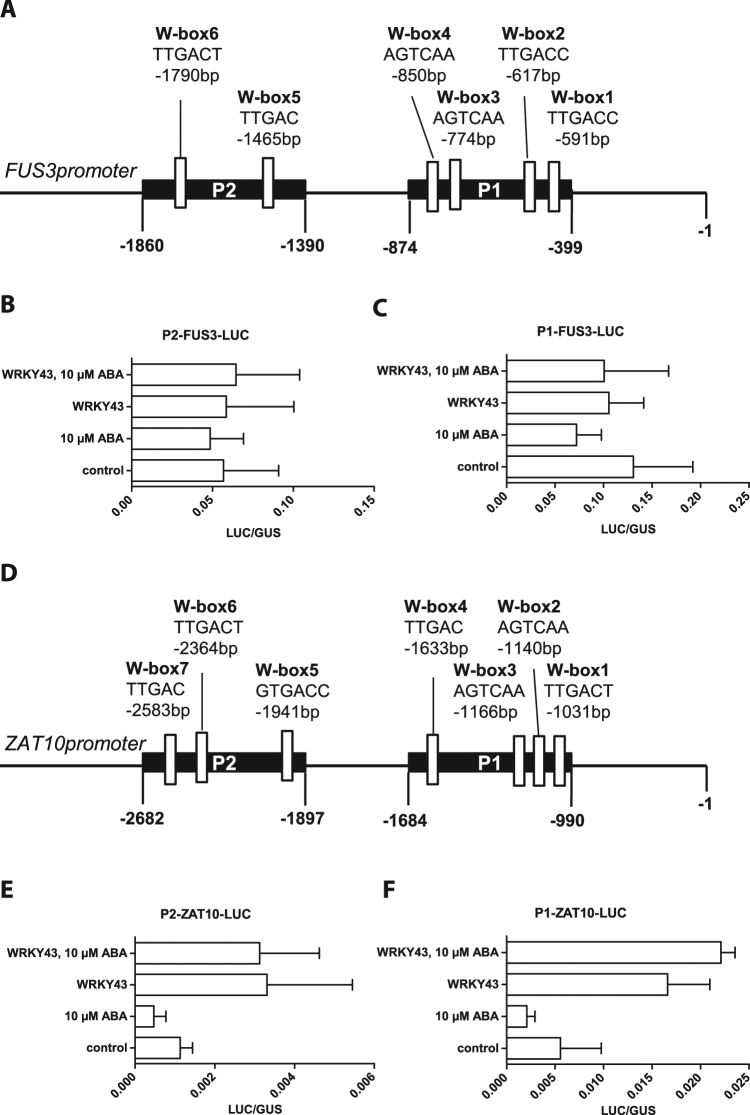
WRKY43 binds directly to *ZAT10* promoter but not to *FUS3* promoter. (**A**) Schematic model of the *FUS3* promoter. W-boxes are marked as white rectangles. Black boxes mark promoter regions. (**B**) Protoplast LUC-assay for *FUS3* promoter regions 2. (**C**) Protoplast LUC-assay for *FUS3* promoter regions 1. Data are shown as bar charts of the ratio of GUS and LUC values (mean ± SE). Promoter-LUC construct was expressed in *A*. *thaliana* wild-type (Col-0) protoplast alone as a control or supplemented with 10 µM ABA and WRKY43 transcription factor. GUS expression was used as an expression control. (**D**) Schematic model of the *ZAT10* promoter. W-boxes are marked as white rectangles. Black boxes mark promoter regions. (**E**) Protoplast LUC-assay for *ZAT10* promoter regions 2. (**F**) Protoplast LUC-assay for *ZAT10* promoter regions 1. Data are shown as bar charts of the ratio of GUS and LUC values (mean ± SE). Promoter-LUC construct was expressed in *A*. *thaliana* wild-type (Col-0) protoplast alone as a control or supplemented with 10 µM ABA and WRKY43 transcription factor. GUS expression was used as an expression control.

To further study differences in regulation of *FUS3* and *ZAT10*, we determined whether WRKY43 could bind directly to *ZAT10* and *FUS3* promoter regions. A LUC-reporter assay was performed in *Arabidopsis* Col-0 protoplasts (Fig. 6). Promoter fragments with putative W-box sequences of *ZAT10* and *FUS3* genes were cloned in front of a luciferase gene and were transiently expressed in Col-0 protoplasts in combination with and without WRKY43 and ABA treatment. *GUS* expression driven by a 35S-promoter was used as a transformation control.

Neither co-expression of *WKRY43* nor ABA treatment induced LUC expression of *FUS3* promoter fragments, indicating that *FUS3* was not directly regulated by WRKY43. By contrast, LUC activity increased upon co-expression of *WRKY43* and *ZAT10* promoter fragments. ABA treatment did not further induce LUC activity. Taken together, the data indicated that WRKY43 directly regulated *ZAT10* expression.

## Discussion

*WRKY43* was primarily expressed during seed maturation, but seed dormancy and embryo development were not affected. Microarray experiments with ABA-treated Ler-0 and *wrky43-1* seeds revealed a down-regulation of SSPs, *FUS3* and *ZAT10* expression in the *wrky43-1* mutant. *WRKY43* mRNA accumulated during the early to mid-seed maturation phase, when the accumulation of storage compound begins^56^. Because FUS3 regulated SSP expression in an ABA-dependent fashion^12,16^, WRKY43 likely influenced the expression of SSPs indirectly via FUS3. Whereas the *FUS3*-promoter region contained 8 putative W-boxes, promoter regions of SSPs contained only very few or no W-boxes. Promoter-LUC-assay also revealed that WRKY43 could not directly regulate *FUS3* gene expression. Therefore, the effects of WRKY43 on *FUS3* transcript levels were possibly mediated via an additional signalling element or via histone modification^57^.

FUS3 positively modulated ABA levels, although *fus3* mutants displayed no change in ABA sensitivity during seed germination, in contrast to *abi3* mutants^15,58–60^. Nevertheless, completely ABA-insensitive were the *fus3-2/abi3-1* double mutants, whereas the single weak *abi3-1* mutant displayed only a moderate ABA phenotype. Therefore, the ABA sensitivity might primarily be regulated by the LAFL member ABI3, whereas FUS holds only a minor role by positively regulating ABI3^61^. Notably, other members of the LAFL network (ABI3, LEC1, LEC2) were not differentially expressed in *wrky43-1* mutant seeds, suggesting that the earlier expression for *LEC1* and *LEC2* during seed stages 4-6 and the later one for *ABI3* during seed stages 9-10 precluded a regulatory hub, whereas *FUS3* and *WRKY43* expression levels both peaked in seed stages 7 and 8^62^.

Salinity reduces water availability for plant use. High salt levels hinder water absorption, inducing physiological drought in the plant. With a high salt concentration, the soil may contain adequate water, but plant roots are unable to absorb it because of the unfavourable osmotic pressure, which is the osmotic or water-deficit effect of salinity. Plants are generally most sensitive to salinity during germination and early growth. The Natural Resources Conservation Service (NRCS) classifies soils with an electrical conductivity (EC) above 4 dS/m as saline, a value that corresponds to approximately 44 mmol salts per litre. Crops vary in their tolerance to salinity and some may be adversely affected at ECs less than 4 dS/m. For example, peach is salt-sensitive, whereas cotton is more salt tolerant^63^. Wild-type *Arabidopsis* seeds displayed reduced germination rates at 50 mM NaCl in the medium, whereas *wrky43-1* mutant seeds germinated at 100% up to 100 mM salt, thereby doubling the soil salinity up to which these plants could germinate without deficit.

The physiological basis for the effects of WRKY43 on stress tolerance and germination might be explained by the changes in fatty acid patterns of seed oil and membrane phospholipids. Loss of *WRKY43* significantly increased seed omega-3 and omega-6 fatty acid content, whereas the total fatty acid content remained unchanged. These findings correlated well with the increased tolerance of *wrky43-1* mutant seeds to cold, salt and osmotic challenge during germination. Plants acclimate to abiotic stress by remodelling membrane fluidity by changing levels of unsaturated fatty acids, which occurs primarily by regulating the abundance and/or activity of fatty acid desaturases through transcriptional and posttranscriptional mechanisms^64^. Phenotypes of the *wrky43-1* mutant resembled phenotypes that overexpressed plastid localized fatty acid desaturases *FAD5*, *FAD6*, *FAD7*, or *FAD8* and ER localized *FAD2* or *FAD3* and had increased tolerance to cold, salt and osmotic stress^53,54,65,66^. This resemblance in phenotypes suggested a regulation of FAD expression or activity through WRKY43. No significant change of FAD transcript levels was detected in green seeds (10 DAF) of the *wrky43-1* mutant and WRKY43 overexpression line. The effects of WRKY43 on fatty acid desaturation could have also occurred at a much earlier stage of seed filling, resulting in a changed fatty acid composition of storage oils that are mobilized for the synthesis of membrane phospholipids during seed germination, thereby temporally uncoupling the presumed effects of WRKY43 on FAD transcription from the manifestation of the changed tolerance to stress conditions. Mechanistically, WRKY43 apparently regulates FAD transcription via an ABA/FUS3 signalling module, because FUS3 triggers FAD expression^16–20^. However, our current data indicated posttranscriptional regulation of FADs.

Transcriptional regulation of *ZAT10* by WRKY43 was ABA-independent, because *ZAT10* transcripts were also down regulated in *wrky43-1* mutant siliques (stages 9-10) in the absence of ABA. The *ZAT10* promoter region contained several putative W-boxes, and the promoter-LUC-assay demonstrated a direct regulation of *ZAT10* by WRKY43. Drought, salt, osmotic and cold tolerance are affected by up- and down-regulation of ZAT10^55,67,68^. Thus, WRKY43 may negatively control abiotic stress tolerance by direct regulation of *ZAT10*. Direct regulation of *ZAT10* is further supported by the WRKY transcription factors from wheat, TaWRKY2, and from soybean, GmWRKY54, which confer salt and drought tolerance by regulating *ZAT10* transcription levels in transgenic *Arabidopsis* plants^69,70^.

## Methods

### Plants and growth conditions

*Arabidopsis thaliana* plants were grown for 4-5 weeks in a growth chamber (200 µE m^−2^ s^−1^, 23 °C, 16 h light, 70% relative humidity). In this study, *Arabidopsis thaliana* accession Col-0 and Ler-0 were used. Open reading frame (ORF) overexpression transcription factor collections (ERF#1-4, WRKY#1-2, bZIP) were provided by Cristoph Weiste and Wolfgang Dröge-Laser^46,47^. *Arabidopsis* mutant *wrky43-1* (ET5604) was originally obtained from the Enhacer-trap collection of Rob Martienssen^71^, and Imre Somssich provided a homozygous line. For analysing *wrky43-1* transposon-insertion, total genomic DNA was extracted from whole siliques. PCR was performed using the following primers: WRKY43 genomic_f, WRKY43 genomic_r, insertion specific primer Ds5-2 (Table S1). RT-PCR was performed on total RNA extracted from whole siliques using the same *WRKY43* gene primer pair and with *ACTIN 2* (*ACT2*) as the control using ACT2_f and ACT2_r.

### Plasmid constructs and plant transformation

For localization studies in *N*. *benthamiana*, full length cDNA of WRKY43 (At2g46130) with attB sites was amplified via PCR using the following primer pair: WRKY43._f and WRKY43._r. Amplification products were subcloned in pDONR221 by BP-reaction and finally cloned into destination vectors pXNSG-YFP^72^. Twenty-five-day-old *N*. *benthamiana* plants were used for *Agrobacterium*-mediated transient expression. Overnight cultures of *Agrobacterium* strain GV3101:pmp90RK, containing binary vectors and strain GV3101:pmp90, which contained the silencing inhibitor p19, were combined, diluted to an OD_600_ of 0.8 in infiltration medium (10 mM MgCl_2_; 10 mM MES, pH 5.6; 100 µM Acetosyringon) and incubated at RT for two hours. Two leaves per tobacco plant were infiltrated and grown under continuous light for two days. Confocal microscopy was performed using an inverted DMIRE2 microscope equipped with a Leica TCS SP2 laser-scanning device (Leica, Wetzlar, Germany, www.leica-microsystems.com). YFP fluorescence was detected at 530-600 nm by excitation at 514 nm with an Ar/Kr laser.

For complementation of the *wrky43-1* mutant line, WRKY43 promoter and gene with and without stop codon were amplified by PCR with attB sites using the following primers: WRKY43 promoter_f; WRKY43_r; WRKY43_r stop. PCR fragment was cloned via gateway cloning into an empty gateway-vector with a C-terminal strepII Tag (pXCG-strepII). The constructs were introduced into *Agrobacterium tumefaciens* strain GV3101:pmp90RK and transformed by floral dip method into *wrky43-1 Arabidopsis* mutants. For the RNAi construct, the WRKY43 coding sequence was transferred to p35S-RNAi by gateway cloning. The resulting plasmid was transformed into *A*. *thaliana* Col-0 by *Agrobacterium*-mediated transformation as before.

### Germination assay

After-ripened seeds were sterilized under chlorine gas and stratified for 3 days in the dark at 4 °C. Plates were incubated in plant incubators at 23 °C on 0.5 MS medium supplemented with increasing concentrations of ABA, NaCl or mannitol. Germination was calculated as percentage of radicle emergence. For data evaluation of concentration series, data were normalized and fitted (log(inhibitor) vs. normalized response with variable slope) for determination of IC_50_ values using GraphPad Prism version 5.00 for Windows (GraphPad Software, San Diego, California, USA, www.graphpad.com).

### Water loss assay and root growth assay

For water loss assays, plants were grown for 4 weeks at 23 °C in plant growth chambers. For each measurement, 20 plants were used; rosettes were cut off at the hypocotyl and placed in a large open petri dish in pools of 5. Rosettes were weighed up to 3 h after cutting on an accurate balance. The *ost1-4* mutant (SALK_008068) was used as the control^73^. Statistical analyses were performed with a nonparametric one-way ANOVA (Kruskal-Wallis test; p < 0.05) using GraphPad Prism version 5.00 for Windows (GraphPad Software, San Diego, California, USA, www.graphpad.com). For the root growth assay, seeds were first spread on 0.5 MS plates and incubated for 4 days, and then seedlings were transferred to 0.5 MS plates supplemented with increasing ABA concentrations. Data were analysed after 4 days incubation using the software Root Detection version 0.1.2 (Labutils, Halle, Germany, www.labutils.de). Statistical analyses were performed with a nonparametric one-way ANOVA (Kruskal-Wallis test; p < 0.05) using GraphPad Prism version 5.00 for Windows (GraphPad Software, San Diego, California, USA, www.graphpad.com).

### Imbibition-assay via seed scanning

Seed sizes were determined using a Canonscan 9000F flatbed scanner (Canon, Krefeld, Germany) as described^74^. For the imbibition assay, at least 200 seeds were put into a bag containing clear water and scanned over a time course of 25 h using eight-bit black and white images with 1200 dpi resolution. Seed size was analysed with ImageJ (1.47v) particle analysing software (National Institute of Health, imagej.nih.gov) with an exclusion size of 40.000 to 150.000 µm^2^ to avoid measurement of non-seed material.

### Ruthenium red staining for seed coat mucilage

Dry seeds were imbibed in water for 1 h without shaking and stained using a ruthenium red solution (0.01% w/v). For the EDTA treatment, seeds were incubated with 50 mM EDTA solution under vigorous shaking for 1 h and then stained by ruthenium red solution as described above.

### Scanning electron microscopy

Dry seeds from the wild type (Ler-0) and *wrky43-1* mutant were directly coated with gold for two minutes and examined in an S-3000 N scanning electron microscope (Hitachi High-Technologies Europe, Krefeld, Germany).

### Transmission electron microscopy

*Arabidopsis* siliques 8 dpi were dissected, and green developing seeds were high-pressure frozen, freeze-substituted and embedded in Spurr’s resin as previously described for ultrastructural studies^75^. Ultra-thin 70 nm sections were examined in a JEM1400 transmission electron microscope (JEOL, Freising, Germany) operating at 80 kV, and micrographs were recorded with a FastScan F214 digital camera (TVIPS, Gauting, Germany).

### Quantification of total fatty acid content and fatty acid composition of dry seeds, 8 DAF seeds and leaves

For lipid analysis, all procedures were conducted in glass containers. Seeds (10 mg) were homogenized in 4 ml of chloroform/methanol/glacial acetic acid (1:2:0.1, v/v/v). Seed residues were removed by centrifugation (2 min, 3000 g). For leaf analysis, leaves of 6-week-old plants were ground in liquid nitrogen and dissolved in 4 ml of chloroform/methanol/glacial acetic acid (1:2:0.1, v/v/v). Phase separation of the aqueous and organic phases was achieved by adding 1.5 ml of chloroform and 1.5 ml of H_2_O, mixing and subsequent centrifugation. The organic phases were collected and dried under streaming N_2_. The lipid residues were dissolved in 1 ml of chloroform. Separation of lipid classes (neutral lipids, galactolipids and phospholipids) was achieved using solid phase extraction on a silica column (Bond Elut SI, 100 mg/ml; Agilent). The column was pre-equilibrated with chloroform, lipid extracts were loaded and subsequently fractionated by successive elution as follows: neutral lipids with 8 ml of chloroform, galactolipids with 8 ml of acetone/isopropanol (9:1, v/v), and phospholipids with 8 ml of methanol/glacial acetic acid (9:1, v/v). The resulting organic phases were dried under streaming N_2_. The dried lipids were redissolved in 100 µl of chloroform. Isolation of individual lipid classes was achieved by thin layer chromatography. For phosphoglycerolipids, developing solvents were chloroform/methanol/glacial acetic acid (65:25:8, v/v/v), for galactoglycerolipids, acetone:toluene:H_2_O (90:30:7, v/v/v) and for neutral lipids, petroleum-ether:diethyl-ether:glacial acetic acid (70:30:2, v/v/v). MGDG, DGDG, PtdCho, PtdIns/PtdSer and PtdEtn were identified according to comigration with authentic standards (Sigma) and reisolated for subsequent derivatization and fatty acid analysis^76^.

FAMEs of TLC-separated individual lipids were obtained by transmethylation with 333 µl of toluol/methanol (1:2, v/v) and 167 µl of 0.5 M NaOCH_3_ at room temperature for 20 min. FAMEs were extracted in 100 µl of NaCl and 2 ml of n-hexane, dried under N_2_, and analysed by gas chromatography. FAMEs were identified by comparison with appropriate reference substances^77^.

FAMEs of total lipids from *Arabidopsis* seeds were prepared from 3 mg of seed material by direct transmethylation with methanol containing 2% (v/v) dimethoxypropane and 2.75% (v/v) sulphuric acid. After 1 h at 80 °C, 0.1 ml of 5 M NaCl was added, and FAMEs were extracted with 2 ml of hexane, dried under N_2_, and analysed by gas chromatography. FAMEs were identified by comparison with appropriate reference substances^78^. FAMEs of single *Arabidopsis* seeds were prepared by transesterification with trimethylsulfonium hydroxide and analysed by GC^79^. FAMEs were identified by comparison with appropriate reference substances.

Gas chromatographic analysis was performed using a Shimadzu GC-2010 plus system, coupled with a flame ionization detector, equipped with a capillary 122-2332 DB-23 column (30 m × 0.25 mm; 0.25 µm coating thickness; Agilent). Helium was used as the carrier gas (1 ml · min^−1^). Samples were injected at 220 °C. The temperature gradient was 150 °C for 1 min, 150 °C to 200 °C at 15 °C min^−1^, from 200 °C to 250 °C at 2 °C min^−1^, and 250 °C for 10 min. Data were processed using the Shimadzu GC-2010 plus software. FAMEs were identified according to retention times of authentic reference standards.

### Microarray

Three independent biological experiments were performed for the transcriptome comparison of Ler-0 and *wrky43-1* seeds. Dry seeds, 10 mg, of Ler-0 wild type and *wrky43-1* mutants were incubated in liquid 0.5 MS medium with 2 µM ABA for 4 days. RNA was extracted from these samples using an RNA-hot-borate extraction protocol^80^ followed by a cleanup using a Qiagen RNeasy Plant kit. *Arabidopsis* Affymetrix Aragene 1.0 st array oligonucleotide-based genome arrays were used, and the NASC’s International Affymetrix Service performed the hybridization and washing.

Microarray data were analysed using Bioconductor and R, based on a modified script from ROBIN v 1.2.4 (Lohse *et al*., 2010) using the following parameters: RMA, post hoc p-value cutoff p < 0.01, nestedF multiple testing strategy and a log2-fold-change minimum of 1. Probesets were mapped to genome loci using the Tair10 annotation file. The data set of this microarray study was deposited in Gene Expression Omnibus (GEO) with the series accession number GSE72154 [reviewer access link: http://www.ncbi.nlm.nih.gov/geo/query/acc.cgi?token = uhipqyognhitzix&acc = GSE72154].

### Quantitative real-time PCR

Harvested samples of *Arabidopsis* tissues were frozen in liquid nitrogen and stored at −80 °C. Developing seeds were dissected out of siliques. For exact determination of age, flowers were marked with little tags, and siliques were harvested after defined days. RNA from all seed samples (dry seeds, germinated seeds, developing seeds, whole siliques) was first extracted by the RNA-borate-extraction method followed by cleanup with a Promega SV total RNA isolation kit. From all root, leave and flower tissues, RNA was directly extracted using a Promega kit. DNA was removed via an on-column DNase treatment. BioRAD iScript^TM^ cDNA Synthesis kit was used to generate cDNA from 1 µg of RNA in a total volume of 20 µl. cDNA samples were diluted 1:10 in water, and 2 µl of this cDNA solution was used as template for qPCR with iQ^TM^ SYBR Green Supermix (20 µl reaction mix) on a BioRad iCycler machine according to the manufacturer’s instructions. As controls, 3 over-all-tissues stable expressed genes were used: ASAR1 (At4g02080), PP2AA2 (At3g25800) and At4g12590^81^. All qPCR primers used are listed in Table S2. Data were analysed with qbase + software (Biogazelle, Gent, Belgium, www.qbaseplus.com).

### Protoplast Luciferase Assay

*Arabidopsis* protoplast transformation and luciferase assay was performed as previously described^82^. Promoter fragments were cloned into pSK-vectors with C-terminal luciferase. GUS expression under 35S-promoter was used as a transformation control. GUS and LUC measurements were performed using a microplate reader (luminometer Centro LB960; Berthold Technologies, Bad Wildbad, Germany, www.berthold.com). Data were calculated as ratios of LUC to GUS.

## Electronic supplementary material

Supplemental Dataset 1
