# Supplementary material for: WRKY43 regulates polyunsaturated fatty acid content and seed germination under unfavourable growth conditions
Source: Sci Rep. 2017 Oct 27;7:14235. doi: 10.1038/s41598-017-14695-0 (PMC5660175; doi:10.1038/s41598-017-14695-0)
Supplement: Supplementary file 1 — Supplemental Dataset 1 [file 41598_2017_14695_MOESM1_ESM.doc]

# WRKY43 regulates polyunsaturated fatty acid content and seed germination under unfavorable growth conditions

Katja Geilen^1^, Mareike Heilmann^2^, Stefan Hillmer^3^, Maik Böhmer^1*^


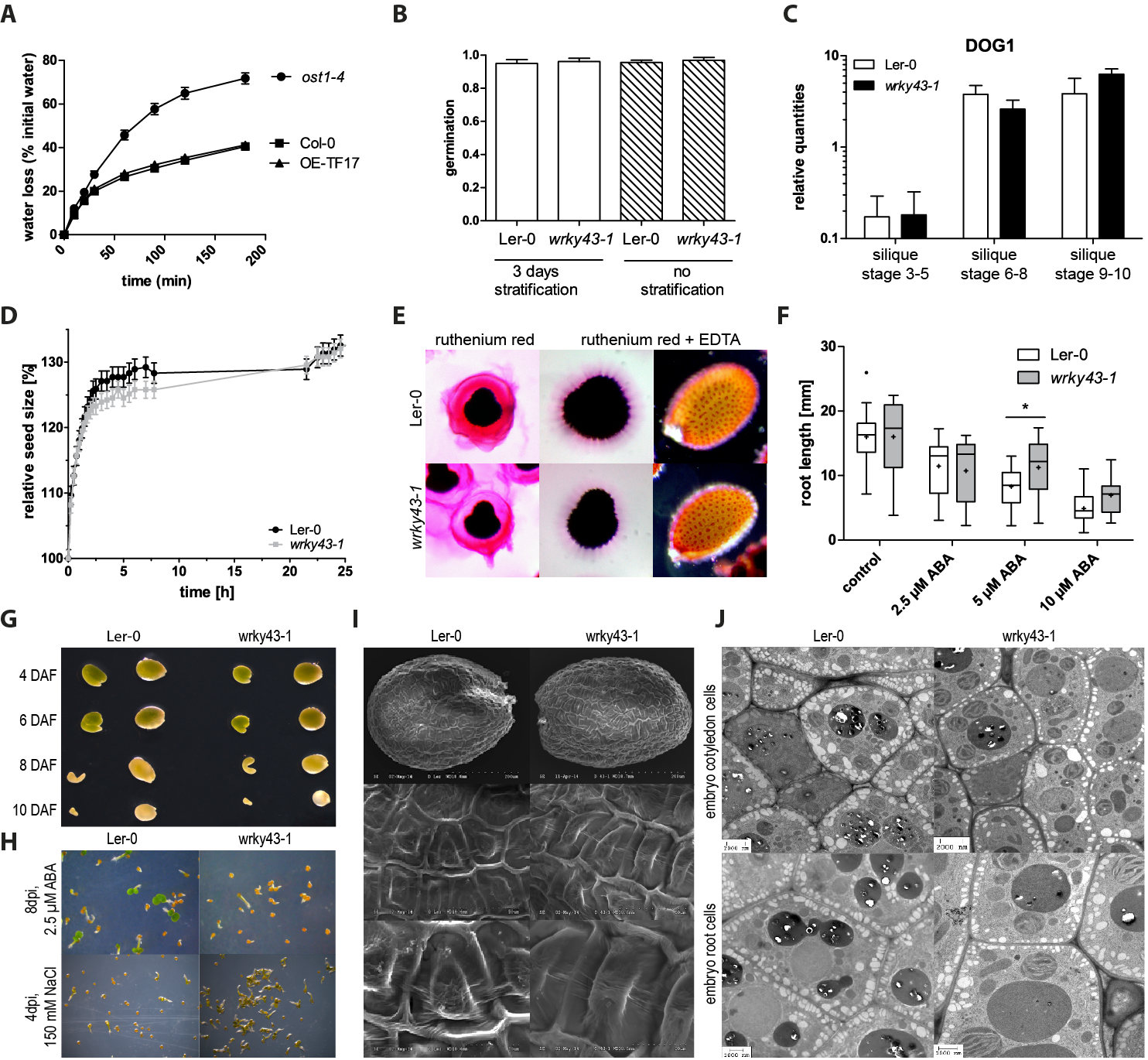


**Figure S 1. Primary dormancy, water loss, ABA dependent root growth, mucilage pro­duction and overall seed morphology are not altered in *wrky43-1*.** (A) Water loss time-course of detached rosette leaves of Col-0 wild type, WRKY43 overexpression (OE-TF17). *ost1‑4* mutant was used as a control. Statistical analysis was performed with a nonparametric one-way ANOVA (Kruskal-Wallis test; p<0.05), revealed no significant differences between Col-0 and OE-TF17 plants. Data are shown as means±SE, n=10. (B) Germination (radicle emergence) of fresh-harvested seeds after 4 days’ incubation on water-agar plates with and without prior stratification for 3 days at 4°C (means±SE, n=3). (C) RT-qPCR analysis of DOG1 transcription levels. RNA was extracted from whole siliques. Data are shown as means±SE, n=3. Statistical analysis was performed with a nonparametric paired t-test (Wilcoxon t-test) (p<0.05) and revealed no significant differences. (D) Relative seed size of water imbibed seeds over 25 h. Data were normalized to the initial seed size (means±SE). (E) Structural comparison of seed coats by mucilage stain with ruthenium red of imbibed seeds. (F) Root length of Ler-0 wild type and *wrky43-1* mutant seedlings after 4 days’ incubation on increasing ABA concentrations. Data are shown as box plots with Tukey whiskers (means±SE). Statistical analysis was performed with a nonparametric t-test (Mann Whitney test) (p<0.05). (G) Morphologies of the developing embryo and the seed coat of wild type (Ler-0) and *wrky43-1* mutant seeds at various stages of seed development. (H) Seed germination of wild type (ler-0) and *wrky43-1* mutant seeds for 8 days on 2.5 µM ABA (upper row) and for 4 days on 150 mM NaCl (lower row). (I) Scanning electron microscopy (SEM) micrographs of seeds of wild type (Ler-0) and *wrky43-1* mutant seeds with details of seed coat micromorphology. (J) Transmission electron microscopy of 8 dpi wild type (Ler-0) and *wrky43-1* embryo cotyledon cells and embryo root cells.

**Figure S 2. Disruption of WRKY43 by an RNAi construct decreases germination on ABA also in the ecotype Col-0.** (A) Germination (radicle emergence) of Col-0 and *RNAi-WRKY43* after-ripened seeds after 3 days’ incubation on increasing ABA concentrations. Data were nor­malized and fitted (log(inhibitor) vs. normalized response) to calculate IC_50_ values (means±SE, n=4).


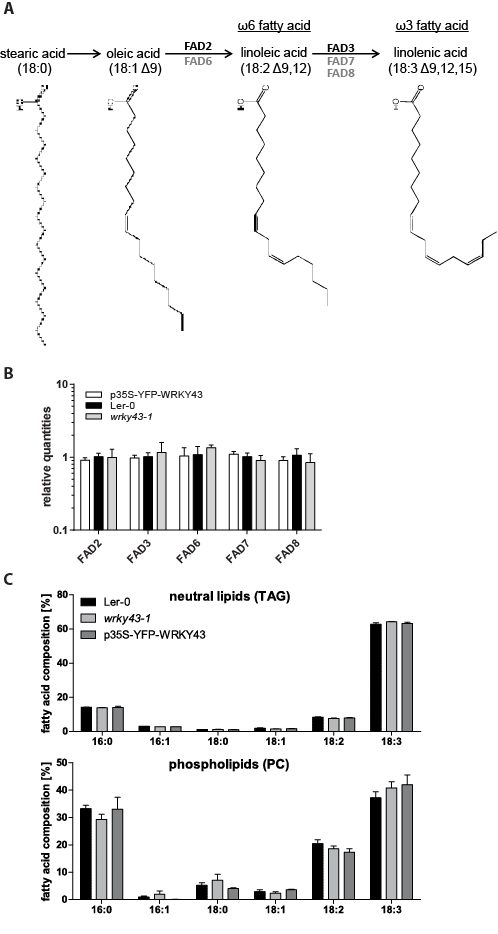


**Figure S 3. FAD regulation by WRKY43.** (A) Model of 18:0 FA desaturation by FADs. (B) Expression profiles of FAD in Ler-0 wild type and p35S-YFP-WRKY43 seeds 10 DAF. RT-qPCR data are shown as bar chart with a logarithmic scale (means±SE, n=3). (C) Triacylglycerol (TAG) and PtdCho fatty acid composition of leaves. Statistical analysis was performed with a One-Way ANOVA (p<0.05) and Bonferroni posttest (data are shown as means±SE, n=4).


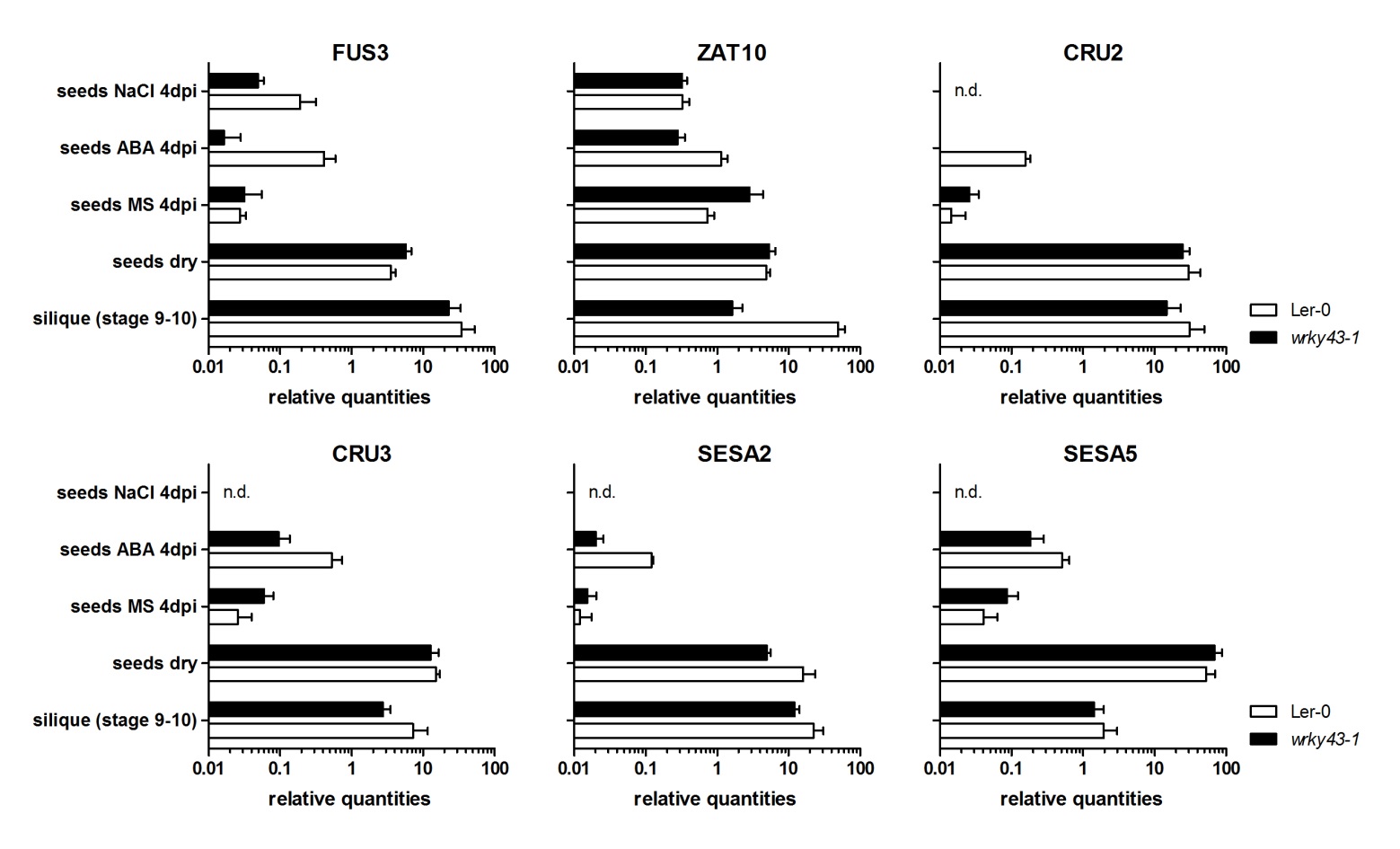


**Figure S 4. Expression analysis of *FUS3*, *ZAT10* and SSPs in *Arabidopsis* seeds at different maturation stages and germination conditions in Ler-0 and *wrky43-1*.** RT-qPCR data are shown as bar chart with a logarithmic scale (means±SE, n=3).

**Table S 1** **Oligonucleotide sequences for genotyping and cloning**

| **Primer name** | **Primer sequence** |
| --- | --- |
| WRKY43 genomic_f | ATGAATGGCCTCGTCGACTC |
| WRKY43 genomic_r | TTAGGTGAACTTAGAGAGGA |
| Ds5-2 | CCGTTTTGTATATCCCGTTTCCGT |
| ACT2_f | gtaagagacatcaaggagaagctctc |
| ACT2_r | ggagatccacatctgctggaatg |
| WRKY43_f | GGGGACAAGTTTGTACAAAAAAGCAGGCTTAATGAAAAATCCGCGATTT |
| WRKY43_r | GGGGACCACTTTGTACAAGAAAGCTGGGTAGGTGAACT TAGAGAGGAA |
| WRKY43 promoter_f | GGGGACAAGTTTGTACAAAAAAGCAGGCTTACCACTGCAGAAAGG TCCA |
| WRKY43_r stop | GGGGACCACTTTGTACAAGA AAGCTGGGTACTATTAGGTGAACTTAGAGAG |

**Table S 2** **Oligonucleotide sequences for quantitative real time PCR**

| **Gene** | **Primer sequence** |  |
| --- | --- | --- |
| ASAR1 At4g02080 | forward  reverse | GCTGTGTTATTATTAAGCCGTAAG  AAAGCTAGGTACGGTTTAAGAC |
| PP2AA2 At3g25800 | forward  reverse | AATCGGTTGTGGAGAAGACG  GCGAAAAACCTGACATCAACAT |
| At4g12590 | forward  reverse | GAGATGAAAATGCCATTGATGAC  GCACCCAGACTCTTTGATG |
| WRKY43 At2g46130 | forward  reverse | Caagaacagcttgtatcccaggag  tcgacagcctctgaacttgcttc |
| DOG1 At5g45830 | forward  reverse | Cgtgagatcgtcgttgagctaa  tctcgagtggatgagtttgca |
| FUS3 At3g26790 | forward  reverse | Gccaaacaacaatagcagaa  tttcttgcttgtataacgtaattg |
| ZAT10 At1g27730 | forward  reverse | tcacaaggcaagccaccgtaag  ttgtcgccgacgaggttgaatg |
| CRU2 At1g03880 | forward  reverse | AGCTTGACCGCAACCTTAGACC  TGTAGCCATTCCTGCCCTTGTG |
| CRU3 At4g28520 | forward  reverse | TGGAGTATGTCAGGCTCAGTGC  AGGAAGCACCATCGCATTCCTG |
| SESA2 At4g27150 | forward  reverse | AACCCTCGAGCGTATGAGAGTG  AAAGACACGATGAGGTGTGGTG |
| SESA5 At5g54740 | forward  reverse | AACGCATCCATCTACCGCACAG  ACCTTGTTGTGGGTTGCTCACG |
